# Facilitation of Dye-Based Quantitative Real-Time Polymerase Chain Reaction with Poly(ethylene glycol)-Engrafted Graphene Oxide

**DOI:** 10.3390/nano13081348

**Published:** 2023-04-12

**Authors:** Khushbu Chauhan, Dong-Min Kim, Eunbin Cho, Dong-Eun Kim

**Affiliations:** Department of Bioscience and Biotechnology, Konkuk University, 120 Neundong-ro, Gwangjin-gu, Seoul 05029, Republic of Korea

**Keywords:** quantitative real-time PCR, DNA binding dye, poly(ethylene glycol)-engrafted nano-sized graphene oxide (PEG-nGO), cycle threshold, melting curve analysis

## Abstract

Quantitative real-time polymerase chain reaction (qPCR) is an important and extensively utilized technique in medical and biotechnological applications. qPCR enables the real-time detection of nucleic acid during amplification, thus surpassing the necessity of post-amplification gel electrophoresis for amplicon detection. Despite being widely employed in molecular diagnostics, qPCR exhibits limitations attributed to nonspecific DNA amplification that compromises the efficiency and fidelity of qPCR. Herein, we demonstrate that poly(ethylene glycol)-engrafted nanosized graphene oxide (PEG-nGO) can significantly improve the efficiency and specificity of qPCR by adsorbing single-stranded DNA (ssDNA) without affecting the fluorescence of double-stranded DNA binding dye during DNA amplification. PEG-nGO adsorbs surplus ssDNA primers in the initial phase of PCR, having lower concentrations of DNA amplicons and thus minimizing the nonspecific annealing of ssDNA and false amplification due to primer dimerization and erroneous priming. As compared to conventional qPCR, the addition of PEG-nGO and the DNA binding dye, EvaGreen, in the qPCR setup (dubbed as PENGO-qPCR) significantly enhances the specificity and sensitivity of DNA amplification by preferential adsorption of ssDNA without inhibiting DNA polymerase activity. The PENGO-qPCR system for detection of influenza viral RNA exhibited a 67-fold higher sensitivity than the conventional qPCR setup. Thus, the performance of a qPCR can be greatly enhanced by adding PEG-nGO as a PCR enhancer as well as EvaGreen as a DNA binding dye to the qPCR mixture, which exhibits a significantly improved sensitivity of the qPCR.

## 1. Introduction

Quantitative real-time polymerase chain reaction (qPCR) is an efficient and commonly availed tool in molecular biology and clinical diagnostics, as the collection of data simultaneously takes place throughout the PCR process [1,2]. This dual performance is achieved by utilizing fluorescence enhancement of fluorogenic DNA binding dyes that reflects the accumulation of amplified double-stranded DNA (dsDNA) [3]. The fluorescence signal measured throughout each cycle is directly proportional to the amount of nucleic acid amplified, thus eradicating the requirement of additional gel electrophoresis for amplicon detection [4]. The most utilized qPCR detection method is based on either fluorescent DNA binding dyes or fluorescently labelled hybridization probe DNA (i.e., TaqMan^®^ probe DNA) utilizing fluorescence resonance energy transfer (FRET) [3,4].

In the dye-based approach, dsDNA intercalating dye allows the quantification of nucleic acid in ‘real-time’, as the fluorescence signal correlates to the amount of dsDNA amplified. However, this fluorescent-dye-based method often generates a false-positive fluorescence signal because dsDNA intercalating dye also binds to any nonspecifically amplified dsDNA [5]. In the probe-based approach, the probe DNA contains fluorescent reporter dye and quencher at its 5′- and 3′-terminus, respectively, in close proximity. In the absence of a target, the reporter and quencher remain close to each other, keeping the probe intact, and there is no emission of fluorescence. In the presence of a target, the fluorogenic probe is cleaved by the 5′ nuclease activity of the Taq DNA polymerase, which results in fluorescence detection [6]. The probe-based approach is specific, but synthesis of a specific probe is required for each sample with a defined length of probe DNA to ensure FRET quenching.

Despite having numerous applications, qPCR is often limited by nonspecific DNA amplifications, due to nonspecific annealing of ssDNAs: primer dimerization and false priming [7]. Since qPCR is regarded as the gold standard for the diagnosis of various infectious diseases, improving qPCR specificity is requisite for the accurate identification of infectious diseases. Several reagents and nano-sized materials, such as organic compounds (dimethyl sulfoxide, betaine, formamide, glycerol) [8,9,10,11,12], buffers [13], protein (bovine serum albumin) [14,15], detergents (Triton X-100 and Nonidet) [16,17], salts (carnitine, tetraalkylammonium, and 1,2-propanediol-trehalose) [17,18,19], and nanoparticles (gold nanoparticles, carbon nanotubes, graphene oxide conjugated gold nanoparticles, quantum dots, etc.) [20,21,22,23,24,25,26,27], have been employed to improve qPCR performance with enhanced efficiency and specificity. Although these additives have demonstrated enhanced efficiency of qPCR to some extent, the underlying mechanism of qPCR specificity enhancement has not been fully explored in terms of molecular interactions between the additive with nucleic acid and reporter fluorescent dye.

Graphene oxide (GO) is a single-layered two-dimensional nanomaterial with a high surface area and various oxygenated functionalities such as carbonyl, carboxyl, epoxide, and hydroxyl groups, rendering water solubility [28,29]. GO binds to single-stranded nucleic acids via hydrogen bonding and π–π stacking between GO and nucleobases, while the affinity for double-stranded nucleic acids is much lower [30,31]. In addition, GO can completely quench adsorbed fluorophores through FRET when the fluorophore-labeled ssDNA is adsorbed onto the GO surface [32]. Upon addition of complementary DNA to the adsorbed ssDNA, the probe DNA is readily desorbed from the GO surface by forming dsDNA, restoring the probe DNA fluorescence [30,32,33]. Thus, GO has been widely utilized as fluorescence-based biosensors for the detection of a wide range of biomolecules based on FRET and real-time monitoring of fluorescently labeled ssDNA probes [34,35,36,37].

During the DNA replication in cells, ssDNA binding proteins (SSBs) play a vital role by preferentially binding to ssDNA and thereby obstructing the reannealing of ssDNAs [38]. In the early cycles of PCR, nonspecific DNA amplicons are generated, due to excess DNA primers forming primer dimers and a falsely primed DNA template. To avoid the annealing between such nonspecific ssDNAs, we previously hypothesized that GO may act as an analogue to SSB proteins, owing to its virtue of preferential binding of ssDNA [39]. However, GO has lower solubility in high salt concentrations (i.e., PCR buffer containing Mg^2+^) and can readily adsorb proteins (i.e., DNA polymerase), restricting the direct utilization of GO in PCR for specificity enhancement [40,41,42]. Thus, we engrafted poly(ethylene glycol) onto the nano-sized GO (nGO) surface with enhanced GO dispersibility in high salt concentration and reduction of the nonspecific adsorption of proteins [39]. This poly(ethylene glycol)-engrafted nanosized graphene oxide (PEG-nGO) has been proven to enhance specificity and fidelity of PCR and loop-mediated isothermal amplification of target genes [39,43]. However, the use of PEG-nGO in PCR was restricted to end-point assay accompanied by electrophoresis of amplicon DNAs [39], and PEG-nGO has not been implicated in a fluorescence-based qPCR system.

Herein, we have expanded the applicability of PEG-nGO in qPCR, which is regarded as the gold standard for the detection of various viral genes. We employed PEG-nGO in qPCR, utilizing the DNA-binding fluorescent dye for detection of influenza viral RNA as a model system. PEG-nGO as a PCR enhancer increased specificity and fidelity of fluorescent-dye-based qPCR by minimizing the generation of nonspecific amplicons, which are primarily caused by primer dimerization and false priming.

## 2. Materials and Methods

### 2.1. Virus Samples and DNA Oligonucleotides

Influenza virus suspension samples (influenza A virus/California/07/2009/H1N1, and influenza B/Massachusetts/02/2012/Yamagata lineage) were provided by the Korea Institute of Radiological & Medical Sciences (Seoul, Republic of Korea). DNA oligonucleotides for detecting the influenza hemagglutinin gene were designed according to the influenza viral RNA segment 4 (hemagglutinin) gene sequence in the GenBank database (H1N1 hemagglutinin gene sequence: GenBank No. NC026433.1, and Yamagata gene sequence: GenBank No. KC892118.1). Primers for detecting influenza hemagglutinin gene were chemically synthesized and purified, using high-performance liquid chromatography (Bionics, Seoul, Republic of Korea).

### 2.2. Chemical Reagents and Enzymes

TransZol Up reagent for RNA extraction was purchased from TransGen Biotech Co., LTD (Beijing, China). SYBR™ Green I, SuperScript™ III One-Step RT-PCR System with Platinum^®^ Taq DNA polymerase kit, and ethidium bromide (EtBr) were purchased from Invitrogen (Carlsbad, CA, USA). EvaGreen^®^ Dye was purchased from Biotium (Hayward, CA, USA). GO (SKU-HCGO-W-175) was purchased from Graphene Supermarket (Calverton, NY, USA) and 6-arm polyethylene glycol (PEG) amine (15 kDa) was purchased from SunBio Inc. (Seoul, Republic of Korea). Chloroacetic acid and N-(3-dimethylaminopropyl-N′-ethylcarbodiimide) hydrochloride (EDC) were purchased from Sigma-Aldrich (St. Louis, MO, USA).

### 2.3. Preparation of PEG-nGO

PEG-nGO was prepared using the previously described protocol [39]. Briefly, GO (2 mg mL^−1^) was fragmented into nanosized-GO (nGO) by tip sonication for 5 h. The addition of sodium hydroxide (2.4 g) and chloroacetic acid (2.0 g) to the nGO suspension (10 mL) with bath sonication for 3 h was carried out for conversion of −OH groups to the −COOH group on the nGO surface, resulting in carboxylated nGO surface (COOH-nGO). The COOH-nGO was neutralized by repeated rinsing with distilled water and filtered by Amicon^®^ stirred cell with 0.2 μm filter membrane (Millipore, Billerica, MA, USA). The 6-arm PEG-amine (2 mg mL^−1^) was added to the COOH-nGO solution and the mixture was bath sonicated for 10 min. Next, 5 mM N-(3-dimethylaminopropyl-N′-ethylcarbodiimide) hydrochloride (EDC) was added to the COOH-nGO solution and stirred overnight at 25 °C. Mercaptoethanol (50 mM) was added for reaction termination and the product (PEG-nGO) was produced by centrifugation at 10,000× *g* for 1 h in 2× phosphate-buffered saline, Supernatant containing PEG-nGO (18 mL as the final volume with a concentration of 25 µg mL^−1^) was saved and stored at 4 °C for subsequent use.

### 2.4. RNA Extraction, Reverse-Transcription qPCR, and Electrophoresis

Total influenza viral RNA was extracted from influenza virus samples (H1N1 and Yamagata strains) using TransZol Up reagent, according to the manufacturer’s instructions. The extracted viral RNA was subjected to one-step reverse-transcription qPCR by using SuperScript™ III One-Step RT-PCR System with Platinum™ Taq DNA Polymerase in real-time thermo cycler Rotor-gene Q system (Qiagen, Hilden, Germany). The reverse-transcription qPCR was carried out in a reaction volume of 25 µL containing viral RNA at varying concentrations, 0.3 µM PCR primers (IFZ A or IFZ B; sequences in Table S1), 1× Taq buffer, 0.2 mM deoxynucleotides, 1.6 mM MgSO_4_, 1.5× EvaGreen, 1 µL Platinum^®^ Taq Mix, and a varied concentration of PEG-nGO (0 to 2.5 µg mL^−1^). The qPCR heat cycling was carried out under the following conditions: cDNA synthesis at 55 °C for 20 min, pre-denaturation at 95 °C for 2 min, 40 cycles of denaturation (95 °C, 30 s), annealing (59 °C, 30 s) and extension (68 °C, 30 s), followed by a final extension at 68 °C for 5 min. The cycle-threshold (Ct) values were determined by the cross-point intersection between the amplification curve and the threshold line, which is 10% of the maximum amplified fluorescence intensity after commencement of the qPCR reaction. Melting curve analysis was performed using the ‘high-resolution melt analyzer’ equipped in the Rotor-gene Q system; amplified DNA samples after qPCR cycles were heated at 95 °C for 90 s and renatured at 40 °C for 5 min. The temperature was then increased from 40 °C to 95 °C with fluorescence change monitoring every 0.5 °C. PCR amplified products were analyzed on 1.5% agarose gel electrophoresis, stained with EtBr and visualized under a UV illuminator (Daehan Scientific Co., Ltd., Wonju-Si, Republic of Korea).

## 3. Results and Discussion

### 3.1. Potential Mechanism of qPCR Facilitation by PEG-nGO

During qPCR, an excess amount of ssDNA primers could generate nonspecific amplicons caused by primer dimerization and false priming. Since dsDNA-binding fluorescence dyes bind to any dsDNA, they tend to generate fluorescence signals even in the presence of nonspecifically amplified DNAs without a target sequence, resulting in false-positive signals. Encouraged by our previous attempt at the beneficial employment of PEG-nGO in PCR [39], we hypothesized that PEG-nGO possessing ssDNA binding ability would adsorb excess ssDNA primers in qPCR and facilitate specific amplification of the target sequence with fluorescence signal enhancement in qPCR, minimizing the false-positive signals due to nonspecific reannealing of ssDNA.

**Scheme 1:**
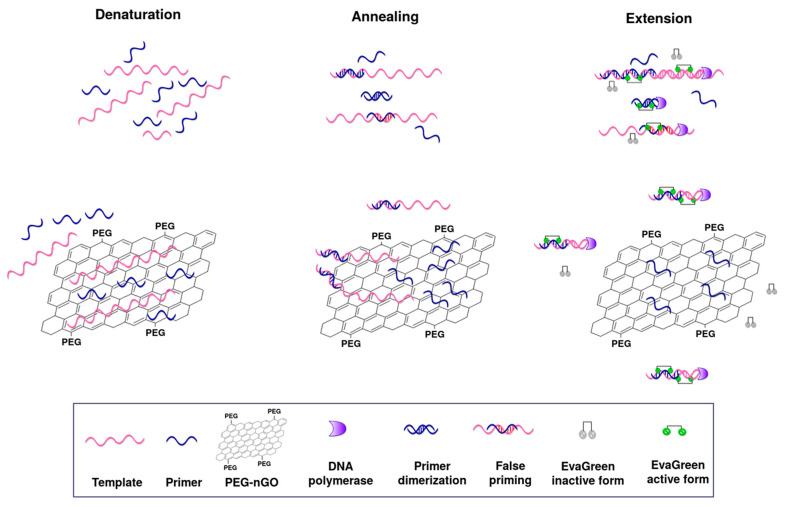
Schematic representation of qPCR facilitation by poly(ethylene glycol)-engrafted nanosized graphene oxide (PEG-nGO). PEG-nGO suppresses nonspecific amplification during thermal cycling of qPCR by adsorbing surplus ssDNA primers, leading to enhanced specificity.

The potential working mechanism of qPCR facilitation by PEG-nGO (dubbed as PENGO-qPCR) with improved specificity is illustrated in Scheme 1. PEGylation on the surface of GO has been reported to significantly reduce protein adsorption [39,44], by which PEG-nGO is expected to adsorb ssDNAs without inducing detrimental protein adsorption on the GO surface. In the qPCR process when primers are in excess, PEG-nGO would adsorb primer DNAs and template ssDNA during the denaturation step, thus preventing primer−dimer formation as well as false priming to the ssDNA template. The annealing step allows ssDNA primers to be hybridized to the target template DNAs, and the extension step enables the synthesis of amplicon DNAs that would be fluorescently responded by dsDNA binding dyes with fluorescence enhancement.

### 3.2. Selection of DNA Staining Dye in PENGO-qPCR

To detect the amplified DNA products in a real-time qPCR analysis, fluorogenic reporters are added into the reaction mixture. These reporters mainly fall into two groups: one is specific DNA probes which are often labeled with fluorophore and quencher, and the other group is dsDNA-binding dyes, such as EtBr, SYBR Green I (SGI), and EvaGreen (EG). Fluorescent DNA stain dyes intercalating dsDNA have been developed for detecting the amplified DNAs in qPCR, in which the non-fluorescent dye becomes highly fluorescent upon binding to DNAs without inhibition of the PCR process. Depending on the mode of fluorescent enhancement upon DNA intercalation, two different fluorescent dyes have been commercially used; SGI DNA staining dye, which preferentially binds to the minor groove of dsDNA with fluorescence enhancement upon DNA base intercalation, and EG DNA staining dye, which specifically bind to grooves of dsDNA with low or no affinity for ssDNA and short DNA fragments, via ‘release-on-demand’ DNA-binding and the fluorescence enhancement mechanism [45,46].

**Figure 1:**
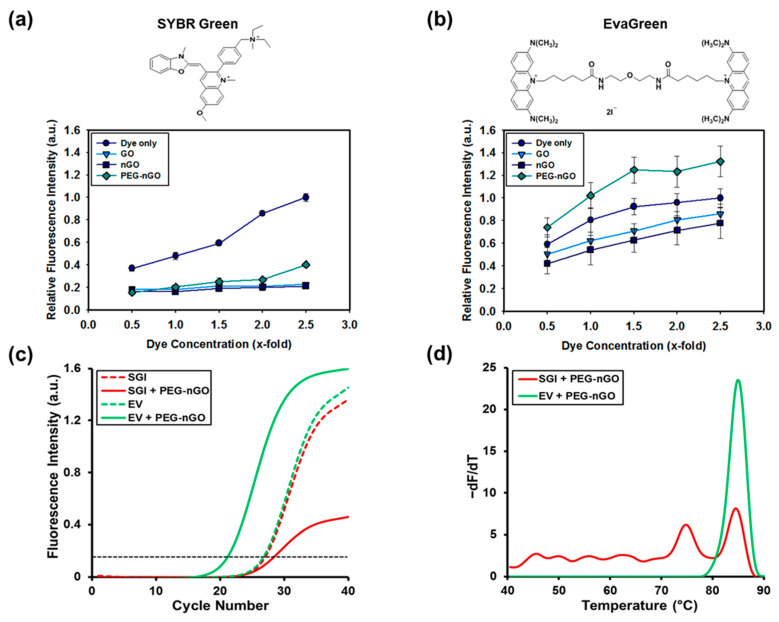
Effect of dsDNA-binding fluorescent dyes on PENGO-qPCR. (**a**) Chemical structure of SYBR Green I (SGI) dye (top panel) and fluorescence intensities of various SGI dye concentrations (0.5-, 1-, 1.5-, 2-, and 2.5-fold) incubated with Plasmid DNA and GO, nGO or PEG-nGO (1 µg mL^−1^), measured at 485 nm and 535 nm as excitation and emission wavelength, respectively. (**b**) Chemical structure of Eva Green (EG) dye (top panel) and fluorescence intensities of various EG dye concentrations (0.5-, 1-, 1.5-, 2-, and 2.5-fold) incubated with plasmid DNA and GO, nGO or PEG-nGO (1 µg mL^−1^), measured at an excitation wavelength of 485 nm and emission wavelength of 535 nm. (**c**) Real-time fluorescence monitoring of target (IFZ B) RNA amplification with SGI dye and EG dye in the presence or absence of PEG-nGO. The cycle threshold (Ct), shown as a horizontal dotted line, is the cycle at which 10% of the maximum amplified fluorescence intensity was employed for signal quantification. (**d**) Melting curve analysis of qPCR-amplified target RNA with SGI dye and EG dye in the presence of PEG-nGO. Melting temperatures (T_m_) were determined from plots of the negative first derivative of change in fluorescence with temperature versus temperature (−dF/dT vs. T).

First, we investigated the compatibility of two commonly used dsDNA binding fluorescent dyes (SGI and EV) in the presence of carbon composites: GO, nGO, and PEG-nGO. Different concentrations (0.5- to 2.5-fold) of both dsDNA binding dyes were incubated with plasmid DNA in the presence of GO, nGO, or PEG-nGO (each carbon composite at 1 µg mL^−1^) and fluorescence was measured while increasing the dye concentrations (Figure 1a,b). There was no increase in fluorescence when plasmid DNA was mixed with GO, nGO, or PEG-nGO along with increasing concentrations of SGI dye (Figure 1a), whereas fluorescence increase was monitored when plasmid DNA was incubated with PEG-nGO and an increasing concentration of EG dye (Figure 1b). It is very likely that the fluorescence of the SGI dye-DNA complex has been quenched through favorable adherent interaction between asymmetrical rings of the SGI and GO surfaces. In contrast, the EG dye contains two monomeric DNA-binding dyes linked by a flexible spacer with a looped conformation that is inactive in DNA binding. When dsDNA is available, the looped conformation shifts to a random conformation that is capable of binding to dsDNA to emit fluorescence via chemical equilibrium, providing a unique ‘release-on-demand’ mechanism. In the presence of PEG-nGO, SGI showed no increase in fluorescence with dsDNA, due to a plausible adsorption of the SGI dye on the surface of PEG-nGO, whereas the EG dye showed an increase in fluorescence with dsDNA, suggesting that the EG dye is compatible with PEG-nGO in reporting dsDNA, due to little adsorption of the EG dye-dsDNA complex onto the PEG-nGO surface.

We next checked the effect of both the SGI and EG dyes on PENGO-qPCR, in which qPCR was carried out for amplification of the target (Influenza B virus; IFZ B) RNA with both SGI and EG as reporter dyes in the presence or absence of PEG-nGO (Figure 1c). The SGI dye system showed lagged and diminished fluorescence signal enhancement with higher Ct and lower fluorescence-intensity values, respectively, in the presence of PEG-nGO than in the absence of PEG-nGO. This result indicates that the SGI dye is not compatible with PENGO-qPCR. On the other hand, the EG dye system showed faster fluorescence enhancement with a lower Ct value in the presence of PEG-nGO, as compared to qPCR without PEG-nGO. This result demonstrates an improved efficiency of EG-dye-based qPCR in the presence of PEG-nGO.

We next examined the effect of DNA stain dyes in the PENGO-qPCR on the melting temperature (T_m_) of PCR products, by measuring the amount of dsDNA as a function of the temperature. IFZ B RNA was amplified using qPCR, and the resulting qPCR-based amplicon DNA was checked by raising the temperature from 40 °C to 90 °C. Derivative melting curves of amplified dsDNA with T_m_ values were displayed by plots of the negative first derivative of fluorescence with change in temperature versus temperature (−dF/dT vs. T) (Figure 1d). The SGI dye showed a specific sharp peak at high temperature, along with nonspecific multiple irregular peaks at low temperatures in the PENGO-qPCR, suggesting the inhibitory effect of PEG-nGO on the performance of SGI-based qPCR. On the other hand, the EG-dye-based qPCR system showed a single sharp peak at high T_m_, suggesting the amplification of a specific target-gene product without nonspecific shorter-amplicon DNAs. Thus, the EG dye system with release-on-demand mode for dsDNA binding is more suitable for the PENGO-qPCR for quantitative monitoring of target-gene amplification with enhanced resolution of fluorescence signals, and was chosen as the amplicon dsDNA reporting dye in the PENGO-qPCR, hereafter.

### 3.3. Effect of PEG-nGO Concentration on qPCR Performance

**Figure 2:**
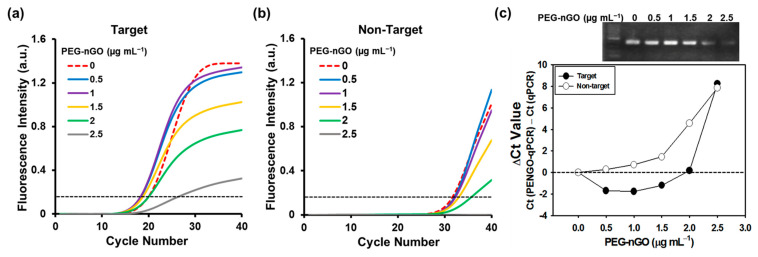
Effect of PEG-nGO concentration on EG-based qPCR. (**a**) Real-time fluorescence monitoring of qPCR-amplified products with target (IFZ B) RNA under various concentrations of PEG-nGO (0, 0.5, 1, 1.5, 2, and 2.5 µg mL^−1^). (**b**) Real-time fluorescence monitoring of qPCR-amplified products with non-target (IFZ A) RNA under various concentrations of PEG-nGO (0, 0.5, 1, 1.5, 2, and 2.5 µg mL^−1^). (**c**) Agarose gel electrophoretic analysis of qPCR-amplified products with target RNA under various concentrations of PEG-nGO (0, 0.5, 1, 1.5, 2, and 2.5 µg mL^−1^) (top panel). ΔCt values of qPCR-amplified products with target and non-target RNA under various concentrations of PEG-nGO (0, 0.5, 1, 1.5, 2, and 2.5 µg mL^−1^). ΔCt values were plotted as the difference between Ct values with PEG-nGO and Ct values without PEG-nGO.

To determine the effect of PEG-nGO concentration on the EG-based qPCR, various concentrations (0 to 2.5 µg mL^−1^) of PEG-nGO were applied to the qPCR reaction for target (IFZ B) and non-target (IFZ A) gene amplification (Figure 2a,b). The Ct values of fluorescence signal enhancement for target-gene amplification in qPCR were decreased with increasing concentration of PEG-nGO (up to 1.5 µg mL^−1^, negative ΔCt values in Figure 2c), as compared to the fluorescence signal traces in qPCR without PEG-nGO (dashed line in Figure 2a), but sharply increased at higher concentrations of PEG-nGO (solid circles in Figure 2c). Since Ct levels are inversely proportional to the amount of target nucleic acid in the sample, the decrease in Ct values corresponds to the increase in amplicon production in qPCR with PEG-nGO. Thus, inclusion of the appropriate amount of PEG-nGO as PCR enhancer significantly improved the efficiency of qPCR, with lower Ct values for a given amount of the target gene.

As shown in Figure 2b, fluorescence signals of qPCR with the non-target viral gene (IFZ A) showed an increase in Ct values with increasing PEG-nGO concentrations, as compared to the fluorescence trace in qPCR without PEG-nGO (dashed line in Figure 2b), with a positively increased Ct-value difference (open circles in Figure 2c). This result indicates an improvement in qPCR specificity in the presence of PEG-nGO. To further confirm the effect of PEG-nGO on qPCR performance, agarose gel electrophoresis was carried out for the post-amplification analysis of PCR products performed with various PEG-nGO concentrations as PCR enhancer (top panel in Figure 2c). Consistent with the qPCR fluorescence signals, increased intensity of target amplicon DNA bands were vividly observed with increasing PEG-nGO concentration (up to 1.5 µg mL^−1^), and were diminished at higher PEG-nGO concentration.

To corroborate the superiority of PEG-nGO as a qPCR enhancer, various concentrations (0 to 2.5 µg mL^−1^) of GO and nGO were employed in qPCR for amplification of the target gene (IFZ B). A fluorescence signal was observed only at 0.5 µg mL^−1^ of GO with a higher Ct value, as compared to without GO, and no fluorescence signals were detected at higher GO concentrations (Figure S1a). Consistent with the fluorescence signals, the post-amplification analysis of PCR products showed amplicon DNA bands in agarose gel at only 0.5 µg mL^−1^ of GO. A similar trend was observed with qPCR carried out in the presence of nGO: fluorescence signals with higher Ct values as well as DNA amplicon bands in the gel electrophoresis were detected only at 0.5 and 1 µg mL^−1^, as compared to qPCR without nGO (Figure S1b). These results were consistent with our previously reported inhibitory effect of GO and nGO on PCR, due to its excess adsorption of DNA primers and DNA polymerase with high affinity [39]. Taken together, we demonstrate that PEG-nGO at an optimal concentration (0.5 to 1.5 µg mL^−1^) can facilitate qPCR performance in terms of specificity and fidelity, which was abrogated at a higher concentration of PEG-nGO. Hereafter, the optimal concentration for PEG-nGO was chosen as 1 µg mL^−1^ as PCR enhancer in the EG-based qPCR system.

### 3.4. Enhanced Specificity of DNA Amplification in PENGO-qPCR

**Figure 3:**
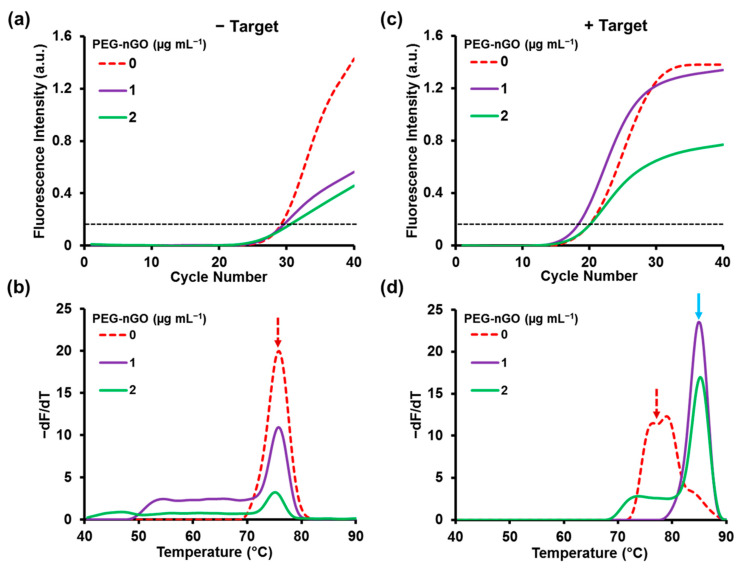
Effect of PEG-nGO on specificity of EG-based qPCR. (**a**) Real-time fluorescence monitoring of qPCR-amplified products in the absence of target RNA (IFZ B) with various PEG-nGO concentrations (0, 1 and 2 µg mL^−1^). (**b**) Melting curve analysis of qPCR-amplified products in the absence of target RNA with various PEG-nGO concentrations (0, 1, and 2 µg mL^−1^). (**c**) Real-time fluorescence monitoring of qPCR-amplified products in the presence of target RNA with various PEG-nGO concentrations (0, 1 and 2 µg mL^−1^). (**d**) Melting curve analysis of qPCR-amplified products in the presence of target RNA with various PEG-nGO concentrations (0, 1 and 2 µg mL^−1^). Red dotted arrow and cyan arrow represent nonspecific- and specific-amplicon dsDNA peak, respectively.

The melting curve analysis of qPCR products with a change in fluorescence as temperature increases, provides useful information for the specificity of DNA amplification during the qPCR process. After thermal cycling in qPCR, amplified dsDNA products are subjected to elevating temperatures for strand dissociation, and a subsequent reduction in fluorescence signal is monitored because of dye detachment from the DNA [47]. Nonspecifically amplified DNAs generated from dimerized primers and false primed amplicons exhibit lower T_m_ values with a broad range of temperatures, whereas specifically amplified DNA amplicons with defined length can be identified as a discrete symmetric peak at higher T_m_ in the melting curve analysis [45,48]. As shown in Figure 3, EG-dye-based qPCR was carried out in the presence or absence of the target gene (IFZ B RNA), with increasing concentrations of PEG-nGO (0 to 2 µg mL^−1^). Fluorescence signals reflecting DNA amplification in qPCR in the absence of the target gene showed an increase in Ct values with increasing amount of PEG-nGO (Figure 3a). The melting curve of the fluorescence signals obtained as nonspecific DNA amplification in the qPCR exhibited a single peak (dashed line in Figure 3b) in the absence of PEG-nGO, making it challenging to distinguish target amplicon from nonspecific amplicons. In contrast, melting curves obtained from the nonspecific DNA amplification in PENGO-qPCR exhibited irregular peaks at lower T_m_, as well as a single peak at a similar T_m_ (dashed arrow in Figure 3b), which decreased in fluorescence intensity with an increasing amount of PEG-nGO. Thus, nonspecifically amplified DNA in qPCR reactions without the target template DNA was diminished with increasing PEG-nGO concentration, suggesting an improved fidelity of qPCR with PEG-nGO.

Next, the fluorescence signals of DNA amplification were obtained in qPCR in the presence of the target gene with increasing PEG-nGO concentrations (Figure 3c). The melting curve of the fluorescence signals obtained as specific DNA amplification in the qPCR without PEG-nGO exhibited an irregular peak, with T_m_ values close to the nonspecific amplicon DNAs (shown as dashed arrow in Figure 3d). However, a sharp peak with higher T_m_ (solid arrow in Figure 3d) was observed with PENGO-qPCR for the target-gene amplification, allowing for the distinguishing of the target amplicon DNA from nonspecifically amplified DNAs. This result suggests that PEG-nGO inhibited the generation of nonspecific amplicon DNAs during EG-dye-based qPCR.

### 3.5. Increased Sensitivity of Target Gene Detection in PENGO-qPCR

**Figure 4:**
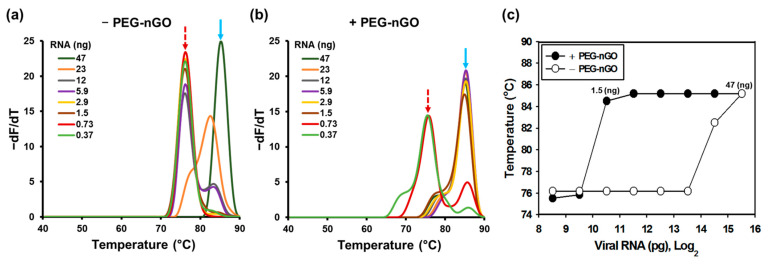
Effect of PEG-nGO on specificity of EG-based qPCR. (**a**) Melting curve analysis of qPCR-amplified products with serially diluted target (IFZ B) RNA (47 ng to 0.37 ng) in the absence of PEG-nGO. (**b**) Melting curve analysis of qPCR-amplified products with serially diluted target RNA (47 ng to 0.37 ng) in the presence of PEG-nGO. Red dotted arrow and cyan arrow represent nonspecific- and specific-amplicon dsDNA peak, respectively. (**c**) Changes in T_m_ values representing amplicon dsDNA peak with various target RNA concentrations in the absence or presence of PEG-nGO (open and closed circles, respectively).

Dye-based qPCR often tends to generate more false-positive signals with less sensitivity and specificity when target nucleic acid is at low copy numbers, as compared to the fluorogenic probes labelled with reporter and quencher dyes [5]. To address whether PEG-nGO is indeed enhancing the sensitivity of dye-based qPCR at low concentration of target nucleic acids, qPCR with EG dye was carried out with serially diluted target nucleic acid (IFZ B) in the presence or absence of PEG-nGO (Figure 4; qPCR fluorescence traces in Figure S2). Derivative melting curves of amplified dsDNA displayed two peaks with differing T_m_ values for serially diluted target nucleic acids in the absence of PEG-nGO (Figure 4a). Single peaks with a higher T_m_ value (85 °C) were only observed with the target nucleic acids at a higher concentration (47 and 23 ng, cyan arrow), and the peak shifted towards the one with lower T_m_ (76 °C), as concentrations of the target RNA decreased gradually (red dotted arrow). Peaks with a lower T_m_ value were thus attributed to nonspecifically amplified dsDNAs in qPCR, with low copy numbers of the target nucleic acid. As shown in Figure 4b, single peaks with higher T_m_ (cyan arrow) in the derivative melting curves of amplified dsDNA were observed at a wider range of target nucleic acid concentrations (47 to 1.5 ng) in the presence of PEG-nGO. As compared with qPCR, the PENGO-qPCR exhibited nonspecific amplicon (red dotted arrow) at much lower concentrations of the target nucleic acid (0.73 and 0.37 ng).

The dynamic range of the specific amplification of the target nucleic acid in qPCR with or without PEG-nGO was compared as a function of the target nucleic acid amount (Figure 4c). Melting curve peaks (T_m_ of 85 °C) representing specific-amplicon DNA were displayed at much lower amounts of the target viral RNA (up to 1.5 ng) in PENGO-qPCR, whereas qPCR without PEG-nGO exhibited specific-amplicon DNA at higher amounts of the target nucleic acid (>47 ng). This result indicates that inclusion of PEG-nGO in the dye-based qPCR significantly enhanced the sensitivity of qPCR for the measurement of low-copy-numbered target nucleic acids. Derivative melting curves of amplified dsDNA with serially diluted non-target RNA (IFZ A) were also compared in the presence or absence of PEG-nGO (Figure S3). In the absence of PEG-nGO, sharply shaped melting curve peaks were observed with different T_m_ values (red and cyan arrows), which are not distinguishable with peaks observed in specific-amplicon DNAs (Figure S3b). In comparison, irregular peaks at lower T_m_ (red dotted arrow) were observed at all the concentrations of non-target RNA in the presence of PEG-nGO, indicating that fluorescence increase in PENGO-qPCR was mainly attributed to nonspecific amplification (Figure S3d).

**Figure 5:**
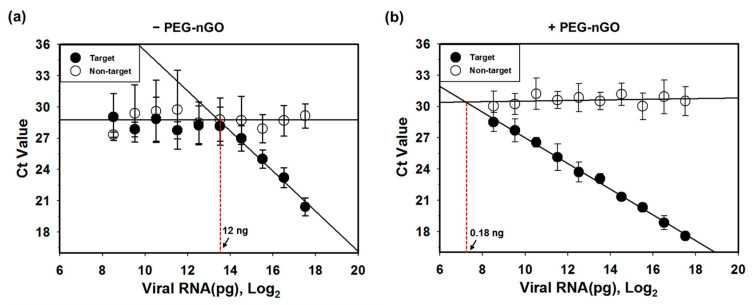
Effect of PEG-nGO on sensitivity of EG-based qPCR. (**a**) Comparison of Ct values of qPCR-amplified products with serially diluted (188 ng to 0.37 ng) target (IFZ B) and non-target (IFZ A) RNA in the absence of PEG-nGO. Red dotted line represents the concentration below which it was difficult to discriminate between target RNA and non-target RNA. (**b**) Comparison of Ct values of qPCR-amplified products with serially diluted (188 ng to 0.37 ng) target and non-target RNA in the presence of PEG-nGO. Red dotted line represents the concentration up to which target and non-target can be distinguished.

Next, to further confirm PEG-nGO as the PCR enhancer in the dye-based qPCR, we compared Ct values of real-time fluorescence traces in qPCR with or without PEG-nGO for the amplification of the serially diluted target (IFZ B) and non-target (IFZ A) viral RNA (qPCR fluorescence traces are shown in Figures S2 and S3, respectively). With the decreasing concentration of viral RNA in qPCR without PEG-nGO, the difference between Ct values of the target and non-target nucleic acids was reduced, and merged at 12 ng of both target and non-target viral RNA (Figure 5a). In contrast, the difference between Ct values of the target and non-target nucleic acids were evident at all amounts of viral RNAs in PENGO-qPCR (Figure 5b). The merging point of Ct values in PENGO-qPCR for target and non-target viral RNAs were obtained at 0.18 ng by extrapolating trends in Ct values. Thus, PENGO-qPCR displayed a 67-fold improved sensitivity in the detection of target nucleic acid (viral RNA) in terms of discernable Ct values, as compared with conventional qPCR.

## 4. Conclusions

The present study indicates the improving effects of PEG-nGO on the specificity, sensitivity, and fidelity of dsDNA-binding dye-based qPCR. Overcoming the limitations of qPCR attributed to nonspecific amplification and false-positive signals is crucial for the specific detection of diseases, as it is recognized as the gold standard for the diagnosis of various infectious diseases. Herein, we demonstrate that PEG-nGO at an optimal concentration facilitates the qPCR with enhanced specificity and sensitivity by suppressing nonspecific amplification. We have evaluated the effect of two commonly utilized fluorescent dsDNA reporter dyes, such as SGI and EV, in which EV was found to be suitable for PENGO-qPCR owing to the scarce adsorption of the EG-dye-dsDNA complex onto the PEG-nGO surface. PENGO-qPCR demonstrated enhanced specificity, with an augmented difference in the T_m_ of target and non-target RNA, as well as improved amplified fluorescent signals for the target RNA, with lower Ct than the non-target RNA, as compared to conventional qPCR. In addition, PEG-nGO enhanced the sensitivity of EG-based qPCR 67-fold, compared to the conventional qPCR, enabling it to distinguish between target and non-target even at low concentrations of the target nucleic acid. PEG-nGO minimizes nonspecific amplification such as primer dimerization and erroneous priming by adsorbing excess primers in early cycles of qPCR, thus leading to qPCR facilitation with improved specificity and sensitivity. We suggest that high specificity and sensitivity of PENGO-qPCR makes it practically applicable in viral gene detection.

## Data Availability

Data that support the findings of this study are available within the article and Supplementary Material.

## Supplementary Materials

The following supporting information can be downloaded at https://www.mdpi.com/article/10.3390/nano13081348/s1, Table S1: Oligonucleotides used in the study; Figure S1: Real-time fluorescence monitoring of target RNA (IFZ B) amplification with GO and nGO; Figure S2: Real-time fluorescence monitoring of qPCR-amplified products with serially diluted (188 ng to 0.37 ng) target (IFZ B) RNA in the absence of PEG-nGO and in the presence of PEG-nGO; Figure S3: Analysis of qPCR-amplified products with non-target (IFZ A) RNA in the presence or absence of PEG-nGO.
