# Supplementary material for: Facilitation of Dye-Based Quantitative Real-Time Polymerase Chain Reaction with Poly(ethylene glycol)-Engrafted Graphene Oxide
Source: Nanomaterials (Basel). 2023 Apr 12;13(8):1348. doi: 10.3390/nano13081348 (PMC10144433; doi:10.3390/nano13081348)
Supplement: Supplementary file 1 [file nanomaterials-13-01348-s001.zip › nanomaterials-2310085-supplementary.pdf]

nanomaterials MDPI

*Supplementary Materials*

# Facilitation of Dye-Based Quantitative Real-Time Polymerase Chain Reaction with Poly(ethylene glycol)-Engrafted Graphene Oxide

**Khushbu Chauhan, Dong-Min Kim, Eunbin Cho and Dong-Eun Kim ***

Department of Bioscience and Biotechnology, Konkuk University, 120 Neundong-ro, Gwangjin-gu, Seoul 05029, Republic of Korea
* Correspondence: kimde@konkuk.ac.kr

**Table S1.** Oligonucleotides used in the study.

| Primer | | Amplicon size (bp) | Sequence (5'-3') |
|---|---|---|---|
| Influenza B (IFZ B) | F | 391 | CTC CAT AGA GGT TCT TCA TTT GGG T |
| Influenza B (IFZ B) | R | 391 | ACA CCT TCT GCG AAA GCT TCA ATA C |
| Influenza A (IFZ A) | F | 193 | TTC AGA CAA TGG AAC GTG TTA CCC |
| Influenza A (IFZ A) | R | 193 | AGA AGC TTT TTG CTC CAG CAT GAG |

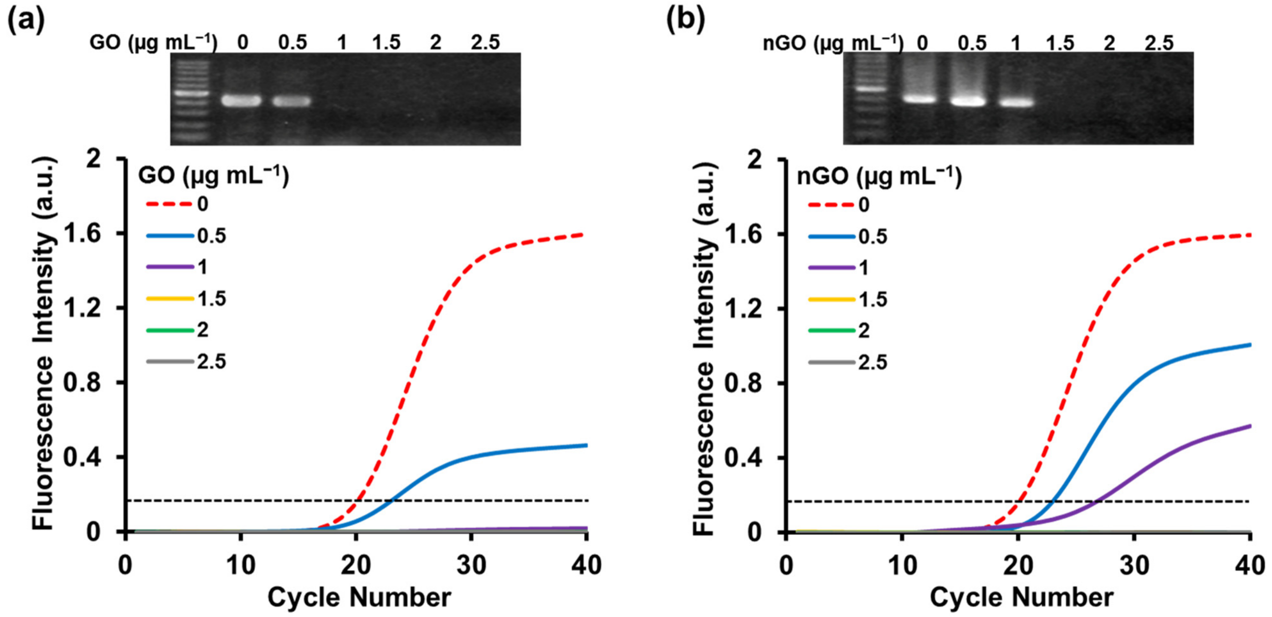


**Figure S1.** Real-time fluorescence monitoring of target RNA (IFZ B) amplification with GO and nGO. **(a)** Agarose gel electrophoretic analysis (top panel) and fluorescent amplification signals with various concentrations of GO (0, 0.5, 1, 1.5, 2, and 2.5 μg mL$^{-1}$). **(b)** Agarose gel electrophoretic analysis (top panel) and fluorescent amplification signals with various concentrations of nGO (0, 0.5, 1, 1.5, 2, and 2.5 μg mL$^{-1}$).

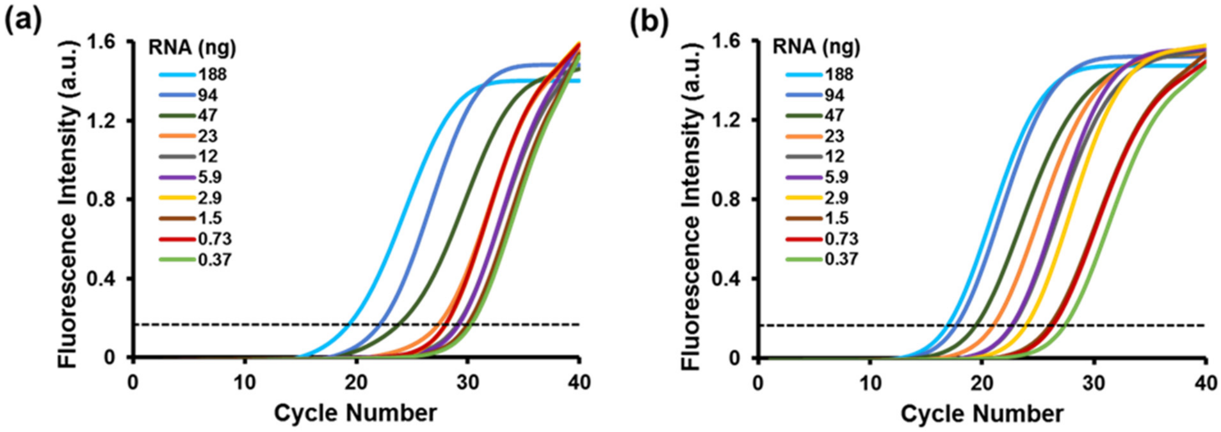


**Figure S2.** Real-time fluorescence monitoring of qPCR-amplified products with serially diluted (188 ng to 0.37 ng) target (IFZ B) RNA **(a)** in the absence of PEG-nGO and **(b)** in the presence of PEG-nGO.

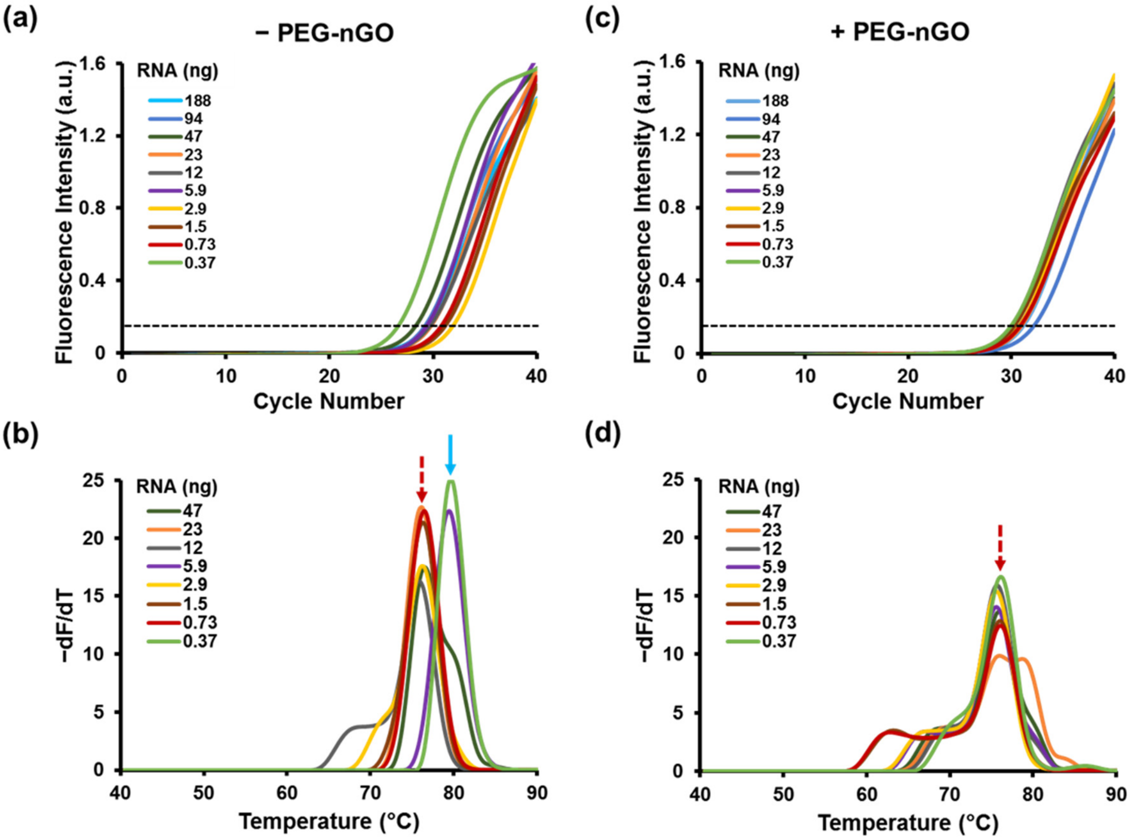


**Figure S3.** Analysis of qPCR-amplified products with non-target (IFZ A) RNA in the presence or absence of PEG-nGO**. (a)** Real-time fluorescence monitoring of qPCR-amplified products with serially diluted (188 ng to 0.37 ng) non-target RNA in the absence of PEG-nGO. **(b)** Melting curve analysis of qPCR-amplified products with serially diluted (47 ng to 0.37 ng) non-target RNA in the absence of PEG-nGO. **(c)** Real-time fluorescence monitoring of qPCR-amplified products with serially diluted (188 ng to 0.37 ng) non-target RNA in the presence of PEG-nGO. **(d)** Melting curve analysis of qPCR-amplified products with serially diluted (47 ng to 0.37 ng) non-target RNA in the presence of PEG-nGO. Red dotted arrow and cyan arrow represents nonspecific and specific amplicon dsDNA peak, respectively.
